# A Pocket Text-Book of Histology and Pathology

**Published:** 1900-04

**Authors:** 


A Pocket Text-Book of Histology and Pathology. By John Benjamin Nichols, Demonstrator of Histology, Medical Department Columbian University, Washington, D. C , and Frank Palmer Vale, Demonstrator of Anatomy and Instructor of Surgery, College of Physicians and Surgeons, Columbia University, New York. Series edited by Bern B. Gallaudet, M. D. Illustrated with 213 engravings. Philadelphia and New York: Lea Brothers & Co. 1900.

This volume presents compactly, clearly, authoritatively and at a very moderate price, the essentials of two important subjects which illuminate each other. The normal structure of the tissues is a standard by which morbid changes can be recognised and measured. Pathology steps in after histology, and regarding each disease as an entity, describes its nature, signs, course and effects, thus yielding an understanding indispensable as a prerequisite to the practice of medicine. Drs. Nichols and Vale have summarised these two sciences in their interrelations, and have presented a manual which is handsomely illustrated, thoroughly up-to-date, and serviceable alike to the practitioner and the student.

The part on normal histology is written entirely by Dr. Nichols and is an excellent exposition of the facts clearly established regarding one of the most important of medical sciences. The author does not take up the consideration of practical methods, but wisely refers to the numerous special works on that subject. The chapters on the cell reproduction and development are carefully prepared and accompanied by illustrations alike beneficial and illustrative. In fact one of the strong points of the work is the good judgment shown in the selection of illustrations. They are all excellent and point out what the author intends they shall stand for. Nothing can be clearer than the plate on page 35, showing a diagramatic representation of the stages of karyokinesis, after Flemming, or the diagramatic sketch of the human kidney, on page 159. The text is well written, rhetoric is well adapted to the class of readers for which this series is intended and no doubt will become a popular series of text-books.

The part on pathology is written by Dr. Vale, who divides it into general pathology in which he treats of the pathology of the blood and circulation, and the pathology of nutrition. Part two deals with special pathology as the diseases of the blood, circulatory system, respiratory organs, gastro-intestinal tract, urinary organs and the nervous system. Here again, as in the part on histology, the illustrations are wisely chosen, particularly the appearances of the corpuscles in circulation, and the spinal cord sections in the chapter on Diseases of the spinal cord.

The authors do not profess to do more than give a résumé of the knowledge of these two subjects and they have succeeded admirably.

The book is handsomely bound, in fact is an ideal binding—bright, durable and catchy. The paper is first-class and the press work up to Lea Brothers' best. W. C. K.

